# Drug Repositioning For Allosteric Modulation of VIP and PACAP Receptors

**DOI:** 10.3389/fendo.2021.711906

**Published:** 2021-11-18

**Authors:** Ingrid Langer, Dorota Latek

**Affiliations:** ^1^ Institut de Recherche Interdisciplinaire en Biologie Humaine et Moléculaire (IRIBHM), Faculty of Medicine, Université libre de Bruxelles, Brussels, Belgium; ^2^ Faculty of Chemistry, University of Warsaw, Warsaw, Poland

**Keywords:** vasoactive intestinal polypeptide, pituitary adenylate cyclase activating polypeptide, neuropeptides, G protein-coupled receptors, virtual screening, molecular dynamics, ticagrelor, allosteric modulator

## Abstract

Vasoactive intestinal peptide (VIP) and pituitary adenylate cyclase-activating polypeptide (PACAP) are two neuropeptides that contribute to the regulation of intestinal motility and secretion, exocrine and endocrine secretions, and homeostasis of the immune system. Their biological effects are mediated by three receptors named VPAC1, VPAC2 and PAC1 that belong to class B GPCRs. VIP and PACAP receptors have been identified as potential therapeutic targets for the treatment of chronic inflammation, neurodegenerative diseases and cancer. However, pharmacological use of endogenous ligands for these receptors is limited by their lack of specificity (PACAP binds with high affinity to VPAC1, VPAC2 and PAC1 receptors while VIP recognizes both VPAC1 and VPAC2 receptors), their poor oral bioavailability (VIP and PACAP are 27- to 38-amino acid peptides) and their short half-life. Therefore, the development of non-peptidic small molecules or specific stabilized peptidic ligands is of high interest. Structural similarities between VIP and PACAP receptors are major causes of difficulties in the design of efficient and selective compounds that could be used as therapeutics. In this study we performed structure-based virtual screening against the subset of the ZINC15 drug library. This drug repositioning screen provided new applications for a known drug: ticagrelor, a P2Y12 purinergic receptor antagonist. Ticagrelor inhibits both VPAC1 and VPAC2 receptors which was confirmed in VIP-binding and calcium mobilization assays. A following analysis of detailed ticagrelor binding modes to all three VIP and PACAP receptors with molecular dynamics revealed its allosteric mechanism of action. Using a validated homology model of inactive VPAC1 and a recently released cryo-EM structure of active VPAC1 we described how ticagrelor could block conformational changes in the region of ‘tyrosine toggle switch’ required for the receptor activation. We also discuss possible modifications of ticagrelor comparing other P2Y12 antagonist – cangrelor, closely related to ticagrelor but not active for VPAC1/VPAC2. This comparison with inactive cangrelor could lead to further improvement of the ticagrelor activity and selectivity for VIP and PACAP receptor sub-types.

## Introduction

Finding new therapeutic purposes for registered drugs is one of the major directions in modern pharmacology. Associated expense and effort reduction rationalizes the drug development process and minimizes its risk. Although dosage, drug formulation or route of administration still may require adjustments to account for newly discovered pharmacodynamics effects, most of preclinical and clinical tests can be omitted. In principle, no structural modifications are introduced to known drugs while repositioning (1), though such adjustments to a new biological target open another pathway in drug discovery. Many of repurposed drugs were discovered by chance, e.g., well-known thalidomide (2), by making use of observed side-effects. This way, drugs that failed in clinical trials due to insufficient efficacy (3) could be given a second chance (4, 5). Nevertheless, systematic approaches involving screening of known active medicines have also been used (6), e.g., in repositioning of antifungal itraconazole as a new anticancer drug (7). Repositioning of antifungal or antiviral agents for chemotherapy (or reverse) dates back to 1960s (8), when idoxuridine, synthesized and described at first as anticancer (9) demonstrated also the antiherpetic activity (10). In some cases, e.g., SARS-CoV-2, time pressure is another reason for turning to drug repurposing in pharmacotherapy (11).

Strategies that are used for drug repositioning can be classified into two major categories: drug-based and disease-based (12), sometimes including one more category: target-based (13). The drug-based strategy employs either genomic or cheminformatics data. The disease-based strategy employs profiling phenotypic traits related to drugs and this strategy seems to be the most successful so far [59% of discovered cases according to (13)]. Computational methods for drug repositioning involve data mining (incl. text mining or semantic techniques), machine learning, network analysis (12) and virtual screening alone or combined with molecular dynamics (14, 15) or machine learning (16). A crucial step for successful drug repositioning remains information retrieval either from multi-purposes databases or resources (17) or drug repurposing-oriented repositories (18), e.g., SIDER (19) or Broad Institute Drug Repurposing Hub (20).

Drug repositioning focused on G protein-coupled receptors has provided so far new anti-filovirus therapeutics, known previously for their antihistamine activity (21). They were discovered in a high-throughput screening of a library of GPCR antagonists (22). Attempts to repurpose GPCR ligands to modulate GPCRs of a different cellular localization, e.g. intracellular, have also been made (23). Growing data on GPCR-related anticancer therapeutics (24) offers new possibilities for drug repositioning. Well-known tamoxifen – a selective modulator of nuclear estrogen receptor but also an agonist of GPER1, has been recently reported for its activity against hepatocellular carcinomas (25). Other examples of successful library screens against GPCRs are: fenoprofen – a COX-2 inhibitor, identified as a new positive allosteric modulator of melanocortin receptor 3 (26), and lorazepam – a modulator of GPR68, proposed as a new therapeutic for pancreatic cancer (27). A more recent example proposed by docking is omarigliptin, DPP-4 inhibitor, repurposed for A_2A_ receptor (28). A different approach to drug repurposing based on virtual screening provided new inhibitors of the PAR-2 receptor (29) by selecting at first 150 hits, out of which 8 compounds were further selected to be tested in bioassays and 4 compounds finally demonstrated an inhibitory effect.

Vasoactive intestinal polypeptide (VIP) and pituitary adenylate cyclase activating polypeptide (PACAP) are neuropeptides that modulate exocrine and endocrine secretions, smooth cell contractility and regulate the immune system. Effects of the VIP action are mediated through interactions with two homologous receptors: VPAC1 and VPAC2. Effects of the second neuropeptide - PACAP are mediated mainly through another class B receptor - PAC1 (ADCYAP1R1), yet it also binds to VPAC1 and VPAC2. All these three receptors are members of a secretin-like subfamily of G protein-coupled receptors (class B GPCRs) that includes receptors for other peptide hormones: secretin, glucagon, glucagon-like polypeptide 1 and 2, calcitonin, parathormone, corticotropin-releasing factor, and GRF. VIP, PACAP and their receptors are widely distributed throughout the body and are thus involved in the regulation of many physiological processes. In the peripheral nervous system, they are involved in the control of insulin secretion from pancreas and release of catecholamines from adrenal medulla. VIP also acts as a co-transmitter of non-adrenergic, non-cholinergic relaxation of vascular and non-vascular smooth muscles. In CNS VIP and PACAP-mediated signaling contributes to circadian rhythm, anxiety, response to stress, schizophrenia, learning, and memory. Finally, VIP and PACAP act as anti-inflammatory agents controlling innate and adaptive immunity (30, 31). For all the above reasons, VIP and PACAP receptor constitute potential targets for the development of new diagnostics and therapeutics for neuronal, metabolic, and inflammatory diseases as well as cancer.

In this study we performed structure-based virtual screening using a world-widely approved ZINC15 drug library and a validated homology model of inactive VPAC1 (VIPR1) to search for new actives among known drugs. Till recently (32), no X-ray or cryo-EM structure of VPAC1 was available. At present, PDB structures of transmembrane domains of VPAC1 or its homolog PAC1 (33, 34) represent only active conformations of these receptors. For this reason, we generated a homology model of VPAC1 based on another class B GPCR – GLP-1R using GPCRM (35–40), molecular dynamics-based model refinement (38, 39, 41) and model verification and validation based on the area under the receiver operating characteristic curve (AUC ROC) (38, 39, 42). Our VS procedure provided ca. 150 hits for each receptor binding site, out of which six compounds were selected for testing in bioassays. Ticagrelor (43–45) demonstrated antagonistic activity against VPAC receptors with the highest response observed for VPAC2, but its closely related compound – cangrelor did not inhibit neither of them. Ticagrelor is an oral, direct-acting P2Y_12_ antagonist with high lipophilicity (46), while cangrelor, another P2Y_12_ antagonist, is administrated intravenously and rapidly inhibits platelet aggregation (47).

To elucidate details of ticagrelor binding modes and the basis of its receptor subtype selectivity we performed molecular dynamics (MD) simulations. Obtained results were consistent with so far confirmed basis of molecular recognition of other class B GPCRs by their negative allosteric modulators binding to the extrahelical active site located in a hydrophobic environment of a lipid bilayer (48–50). Altogether, our results suggest that ticagrelor acts as a negative allosteric modulator of VIP and PACAP receptors with weak selectivity for the VPAC2 receptor sub-type.

## Results and Discussion

### Structure-Based Virtual Screening - ZINC15 World-Widely Approved Drugs

Recent advances in cryo-EM of class B GPCRs provided new structures of VIP and PACAP receptors and molecular basis of their activation, different from other GPCR classes (51). Till recently, only water soluble ECD domains of these receptors have been determined by X-ray crystallography and released in 2007 and 2010 (52) (PDB ID: 2JOD and 2X57 – PAC1 and VPAC2, respectively). Yet, for complete understanding of mechanism of action of these secretin-like GPCRs also their transmembrane domains must be described in detail. Released so far structures of TM domains of VIP and PACAP receptors included peptide agonists and thus represented only active conformations of these receptors. In contrast, inactive conformations of VIP and PACAP receptors remain to be determined or released.

**Figure 1:**
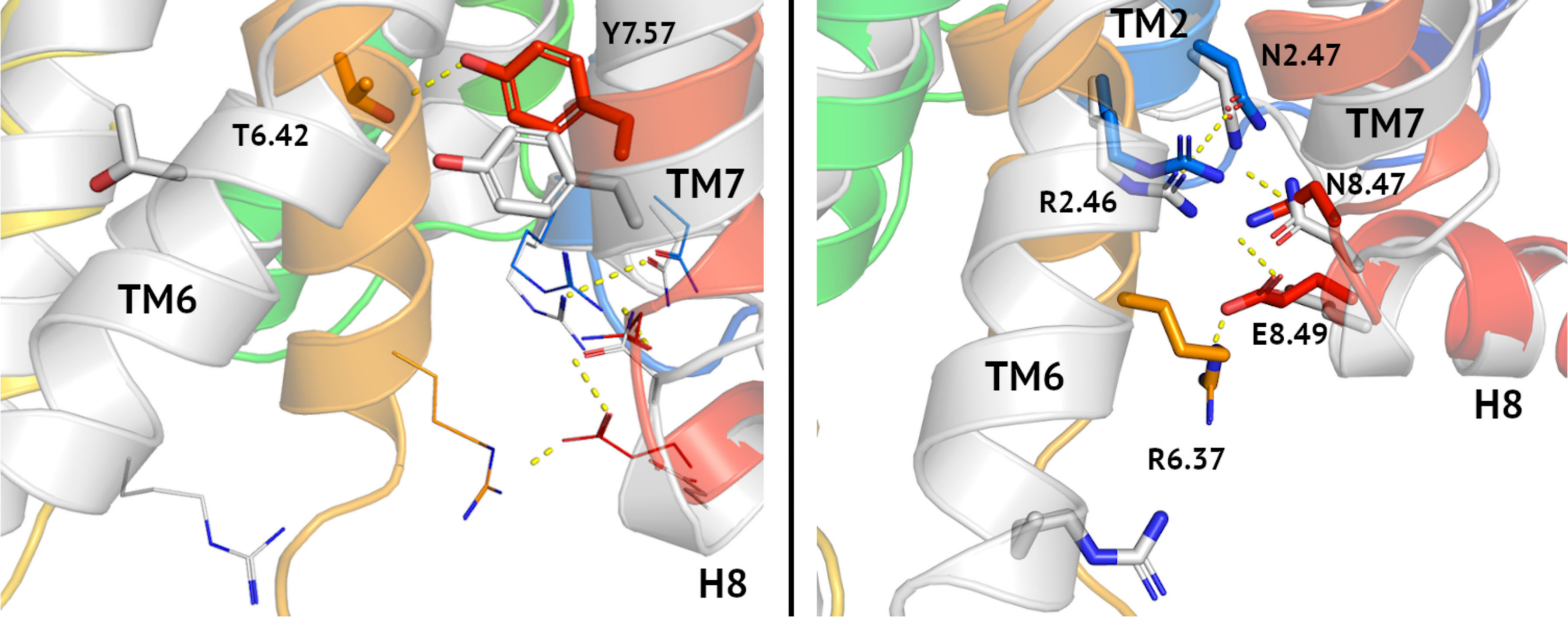
Tyrosine toggle switch in VPAC1. Comparison between inactive (blue-to-red) and active (grey) conformations of VPAC1 in the region of tyrosine toggle switch (left) and the adjacent TM2-TM7-H8-TM6 hydrogen bond network (right). Hydrogen bonds were depicted as yellow dashed lines. Both, the tyrosine toggle switch and the adjacent hydrogen bond network change significantly during the receptor activation, mostly due to the TM6 bending and its counterclockwise rotation. Only N2.47-R2.46 interactions (right) were left in the active conformation of the receptor in this region comparing the inactive one.

Recently, we described homology models of VPAC1 and PAC1 receptors (38, 39, 41) and validated them in virtual screening using DUD-E decoys (38, 39, 42). In **Figure 1** we compared our model of inactive VPAC1 with a PDB structure of active VPAC1 (32). Activation of VPAC1 receptor involves breaking a Thr6.42-Tyr7.57 hydrogen bond [tyrosine toggle switch (53)] due to bending and twisting of TM6. Adjacent residues: Asn2.47, Arg2.46, and Asn8.47, also joined with hydrogen bonds, change their position to a lesser extent during the receptor activation. In contrast, moving of Arg6.37 and Glu8.49 breaks two hydrogen bonds: R6.37-E8.49 and N8.47-E8.49 that are present in an inactive VPAC1 conformation. Negative allosteric modulators of class B GPCRs interact with i.a. Asn8.47 and thus stabilize this intracellular hydrogen bond network of an inactive receptor (49).

The homology model of inactive VPAC1, presented in **Figure 1** and validated by AUC ROC (38, 39, 41), was used in virtual screening against a subset of the ZINC15 compound library including drugs approved world-widely. This subset included drugs approved in major world jurisdictions, including FDA-approved drugs. We focused on two binding sites of VPAC1: orthosteric (TMD and ECD) and allosteric (TMD) – for negative allosteric modulators. So far, only structures of class A GPCRs, not class B GPCRs, included antagonists in their orthosteric binding sites (50). Out of ca. 6800 generated ligand poses we selected up to 150 top-scored poses and manually assigned corresponding compounds to drug classes (see **Supplementary Table S1**). Out of these we selected several drug classes of potential interest based on data already available (**Supplementary Table S2**), and out of this we selected six compounds for pharmacological testing (see **Table 1**). We excluded, e.g., beta-blockers or statins that frequently demonstrate low values of XP-Gscore in VS against various GPCR structures and may act as false-positives, as was observed previously (38, 39).

**Table 1:** Compounds selected by virtual screening for testing in bioassays.

ZINC ID	Name	Drug class	XP-Gscore	VS data set
ZINC000028957444	ticagrelor	P2Y12 receptor antagonist	-9.699^1^	TMD - orthosteric
			-5.029	TMD - allosteric
			-5.254	ECD - orthosteric
ZINC000085537017	cangrelor^2^	P2Y12 receptor antagonist	-9.237	TMD - orthosteric
			-6.958	TMD - allosteric
			-8.884	ECD - orthosteric
ZINC000003918138	zanamivir	Neuraminidase inhibitor	-9.705	ECD - orthosteric
ZINC000009330880	rebamipide	COX activator	-7.971	ECD - orthosteric
ZINC000000001473	flupirtine	NMDAR antagonist	-7.601	ECD - orthosteric
ZINC000001543475	tenofovir	RT inhibitor	-6.422	TMD - allosteric
ZINC000000000507	midrodine	α-AR agonist	-4.001	TMD - allosteric

^1^XP-Gscores computed for one compound but for different binding sites are not comparable in this case, i.e., cannot be used to decide on the compound mechanism of action (orthosteric vs. allosteric). In contrast, XP-Gscores computed for different compounds but for the same binding site are comparable and provide valuable information about the relative fitness of different compounds to the given receptor cavity.

^2^This compound was closely related to ticagrelor and for this reason was selected from VS data sets for testing in pharmacological assays.

### Results From Pharmacological Assays

**Figure 2:**
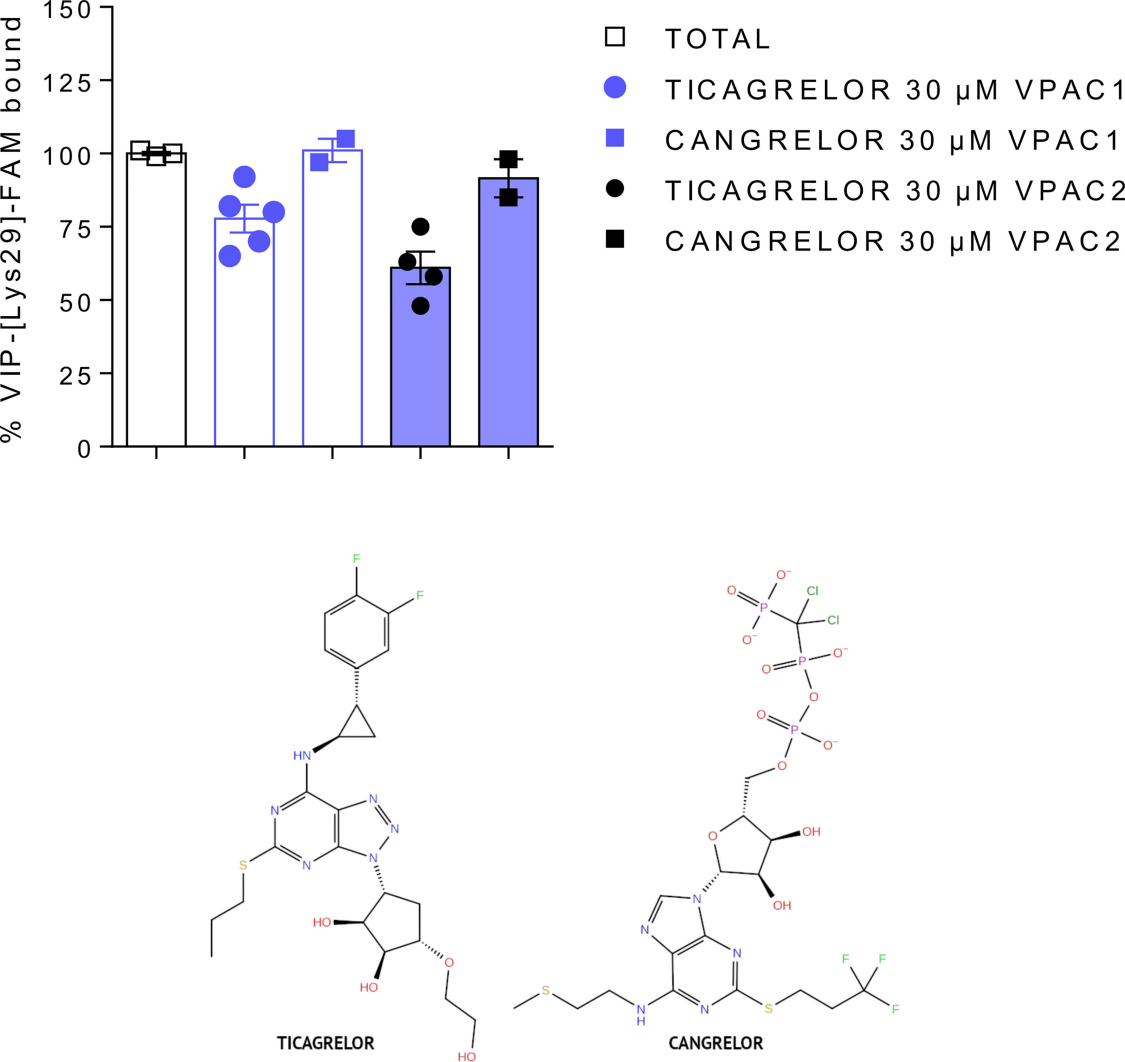
Comparison of the effect of ticagrelor and cangrelor in VIP competition binding assays (top panel). Ticagrelor (closed circles) reduced VIP binding to VPAC1 and VPAC2, while cangrelor (closed squares) had no significant effect. Both compounds share similar scaffolds (bottom panel).

In the first step, we tested selected compounds in VIP competition binding assays. Using carboxyfluorescein-labelled VIP as tracer, we evaluated by FACS analysis the ability of compounds to compete with VIP binding to VPAC1 and VPAC2 receptors. We tested concentrations up to 30 µM (the highest possible concentration due to solubilization concerns) but none compound significantly modified VIP binding except ticagrelor (see **Table 2**, **Figure 2** and **Supplementary Figure S2**). Ticagrelor decreased the specific VIP binding to VPAC1 and VPAC2 receptors by 22% and 39%, respectively (**Table 2**). Competition binding curves were also performed for ticagrelor concentrations up to 30 µM (**Supplementary Figure S11**). The effect of ticagrelor is dose-dependent but as the curves don’t reach a plateau, reliable IC50 values cannot be calculated.

**Table 2:** Results of binding and functional VPAC1/VPAC2 assays for selected compounds.

Tested compound^1^	VPAC1			VPAC2		
	Competition binding assay^2^	Calcium mobilization assay^3^		Competitionbinding assay	Calcium mobilization assay	
	% VIP specific binding	% VIP-induced response	% PACAP38-induced response	% VIP specific binding	% VIP-induced response	% PACAP38-induced response
**tenofovir**	114 ± 5^4^	88 ± 3	89 ± 4	99 ± 1	83 ± 5	nd^5^
**zanamivir**	106 ± 5	84 ± 2	89 ± 1	99 ± 9	85 ± 5	nd
**midrodine**	115 ± 5	85 ± 4	91 ± 4	99 ± 6	86 ± 4	nd
**flupiritine**	127 ± 10	83 ± 4	82 ± 5	109 ± 2	90 ± 7	nd
**rebapimide**	90 ± 5	84 ± 5	82 ± 2	94 ± 4	87 ± 7	nd
**ticagrelor**	78 ± 5	57 ± 6	68 ± 4	61 ± 6	42 ± 6	24 ± 5
**cangrelor**	101 ± 4	101 ± 7	95 ± 5	92 ± 7	103 ± 5	110 ± 8

^1^30 µM concentration of each compound was used.

^2^VIP competition binding assay.

^3^VIP- or PACAP-38-induced intracellular calcium increase in CHO cells expressing VPAC1 or VPAC2 receptors.

^4^Results are the mean ± SEM of at least two independent experiments performed in duplicate.

^5^Not determined.

**Figure 3:**
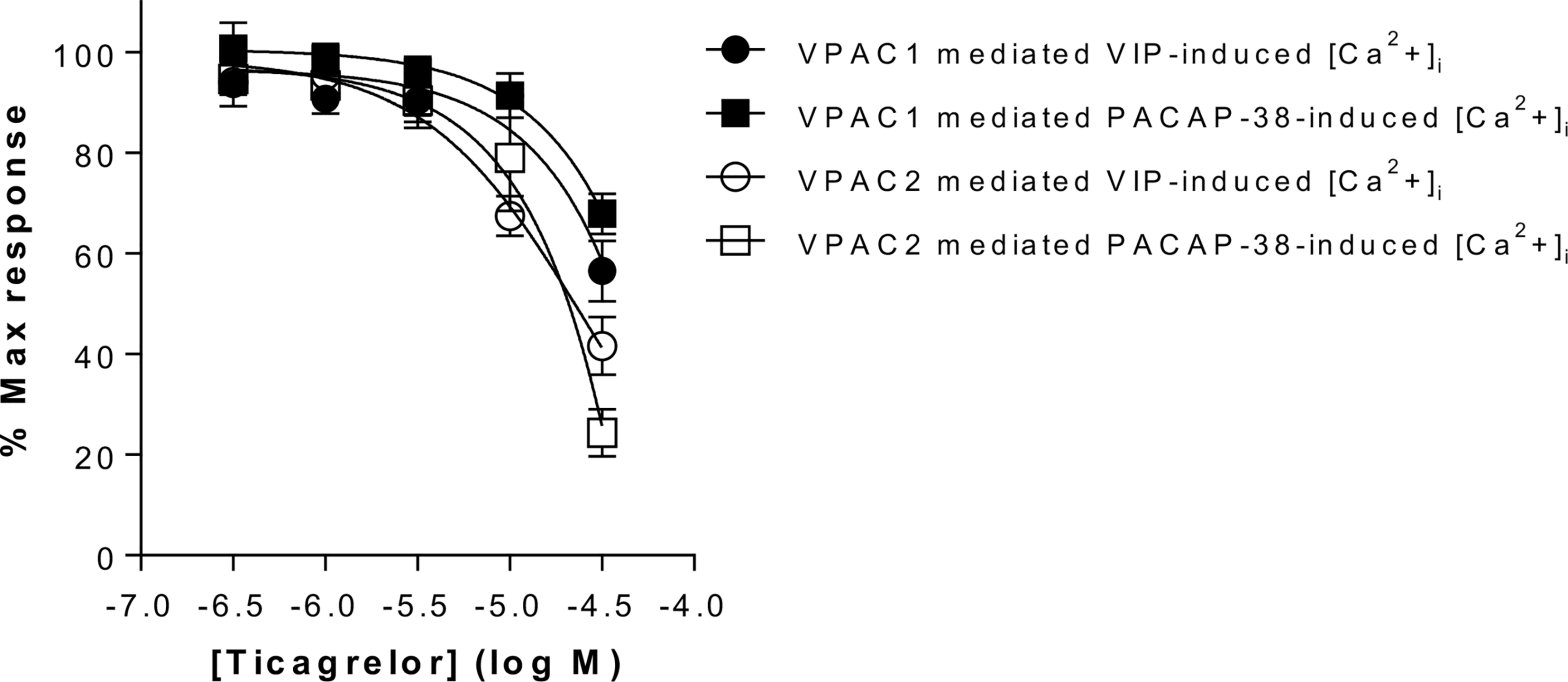
Impact of ticagrelor on VPAC1 and VPAC2 receptors. Inhibition of VPAC1 and VPAC2 activity by ticagrelor was assessed in an aequorin-based calcium-mobilization assay. The response of CHO cells expressing VPAC1 to 3 nM VIP or 10 nM PACAP-38 and of CHO cells expressing VPAC2 to 10 nM VIP or 30 nM PACAP-38 was tested in the presence of increasing concentrations of ticagrelor. Results are the mean ± SEM of at least three independent experiments performed in duplicate.

In the second step, selected compounds were tested using functional assays. We used calcium mobilization functional assays that included cell lines expressing VPAC1 and VPAC2 receptors. Only ticagrelor decreased the VIP- and PACAP-38-induced intracellular calcium increase in cells expressing either VPAC1 or VPAC2 (see **Table 2**, **Figure 3** and **Supplementary Figure S3**). As the inhibitory effect of ticagrelor was partial and did not reach a plateau, reliable IC50 values are difficult to calculate. At best, we can extrapolate that the IC50 values of ticagrelor are of about 50 µM and 20 µM for VPAC1 and VPAC2 respectively. None of the compounds displayed any agonistic activity nor modified the basal activity of the cells expressing each receptor (data not shown).

One compound (ticagrelor) out of six selected by VS demonstrated rather weak but noticeable response in the described above binding and functional assays. We also performed a similarity search [Daylight/Tanimoto descriptors (54, 55)] to find compounds similar to this compound among VS results that could demonstrate a better response in pharmacological assays. We have found several similar compounds (see **Supplementary Table S3**), and we decided to test one additional compound – cangrelor (56), a P2Y12 antagonist closely related to ticagrelor, yet without any significant success (see **Table 2**, **Figure 2** and **Supplementary Figure S1**).

Antagonists of the P2Y_12_ purinergic receptor, ticagrelor and cangrelor, share similar nitrogen-rich aromatic ring systems (43): triazolopyrimidine and purine, respectively. These aromatic rings are connected with small non-aromatic rings: cyclopentanediol and ribose (ticagrelor and cangrelor, respectively). In the latter case (cangrelor), this two-rings system forms an adenosine group, like in ADP - an endogenous agonist of P2Y_12_. Therefore, cangrelor plausibly binds to the same, orthosteric site of P2Y_12_ as ADP in contrast to ticagrelor (57). Ticagrelor and cangrelor differ the most by substituents of non-aromatic rings. Ticagrelor includes a short, slightly polar, hydroxyethyl chain, while cangrelor includes a long, negatively charged chain consisting of three phosphoryl groups separated by a dichloromethyl group. Other differences constitute substituents of aromatic rings. A large and bulky group including 2-difluorophenyl-cyclopropylamine is included in ticagrelor, while in cangrelor it is replaced with a short aliphatic chain with 2-methylsulfanyl-ethylamine. A propylsulfanyl group in ticagrelor is replaced with a trifluoropropylsulfanyl group in cangrelor. These differences in chemical structures of ticagrelor and cangrelor certainly contribute to their different effect on VPAC receptors.

### Ticagrelor Binding Mode to VIP and PACAP Receptors

Ticagrelor was selected from the VS data set generated using the TMD orthosteric site. Yet most probably, it binds to the allosteric TMD site, acting as a negative allosteric modulator (see **Figure 5**) which we concluded based on the below. First, ticagrelor is a highly lipophilic drug in contrast to intravenously administrated cangrelor. Thus, the lipid-facing extrahelical allosteric binding site can be easily reached by it, again in contrast to cangrelor which did not bind to the receptor at all. These different soluble properties of ticagrelor and cangrelor results from the fundamental difference in their chemical structures. A long, negatively charged phosphoryl group in cangrelor, not present in ticagrelor, may prevent its entering to the hydrophobic environment of a lipid bilayer. If cangrelor and ticagrelor demonstrated the same water-exposed, orthosteric binding modes, rather both of them could induce a noticeable response on VPAC1 and VPAC2 but this was not observed.

Although both drugs, ticagrelor and cangrelor, block the activation of the purinergic receptor P2Y_12_ as inverse agonists (57) and thus demonstrate the antiplatelet therapeutic effect, it is still under debate whether ticagrelor is a non-competitive inhibitor of P2Y_12_ (47). Garcia et al. (57) described ticagrelor as non-competitive inverse agonist of P2Y_12_ that binds to a different region of this receptor than cangrelor and endogenous ADP. What is more, they discovered that cangrelor and ticagrelor stabilize different conformations of P2Y_12_ and therefore cangrelor acts as a biased inverse agonist (G_o_α) while the ticagrelor potency is independent of G-alpha subtypes. However, an earlier study of Hoffmann et al. (58) suggested that ticagrelor is a competitive and reversible antagonist of P2Y_12_. In addition to the P2Y_12_ inhibition, ticagrelor has been shown to block equilibrative nucleoside transporter 1 (ENT-1). Although it may also contribute to its antiplatelet activity, a much greater therapeutic effect is associated with its active metabolite (deshydroxyethoxy ticagrelor) (47).

Moreover, an allosteric mode of ticagrelor inhibition of VIP receptors is in agreement with recently released PDB structures of class B GPCRs which we described in (50). Namely, recently determined class B structures included only small molecule agonists in the orthosteric TMD binding site but not inhibitors. An example of an antagonist of class B GPCRs located inside the TMD domain, but not in the region occupied by endogenous peptide ligands, is CP-376395 which blocks the CRF1R activation (PDB id: 4K5Y and 4Z9G) (59). Nevertheless, CP-376395 is a linear-shape molecule and is much smaller than ticagrelor and other class B NAMs, e.g., NNC0640.

**Figure 4:**
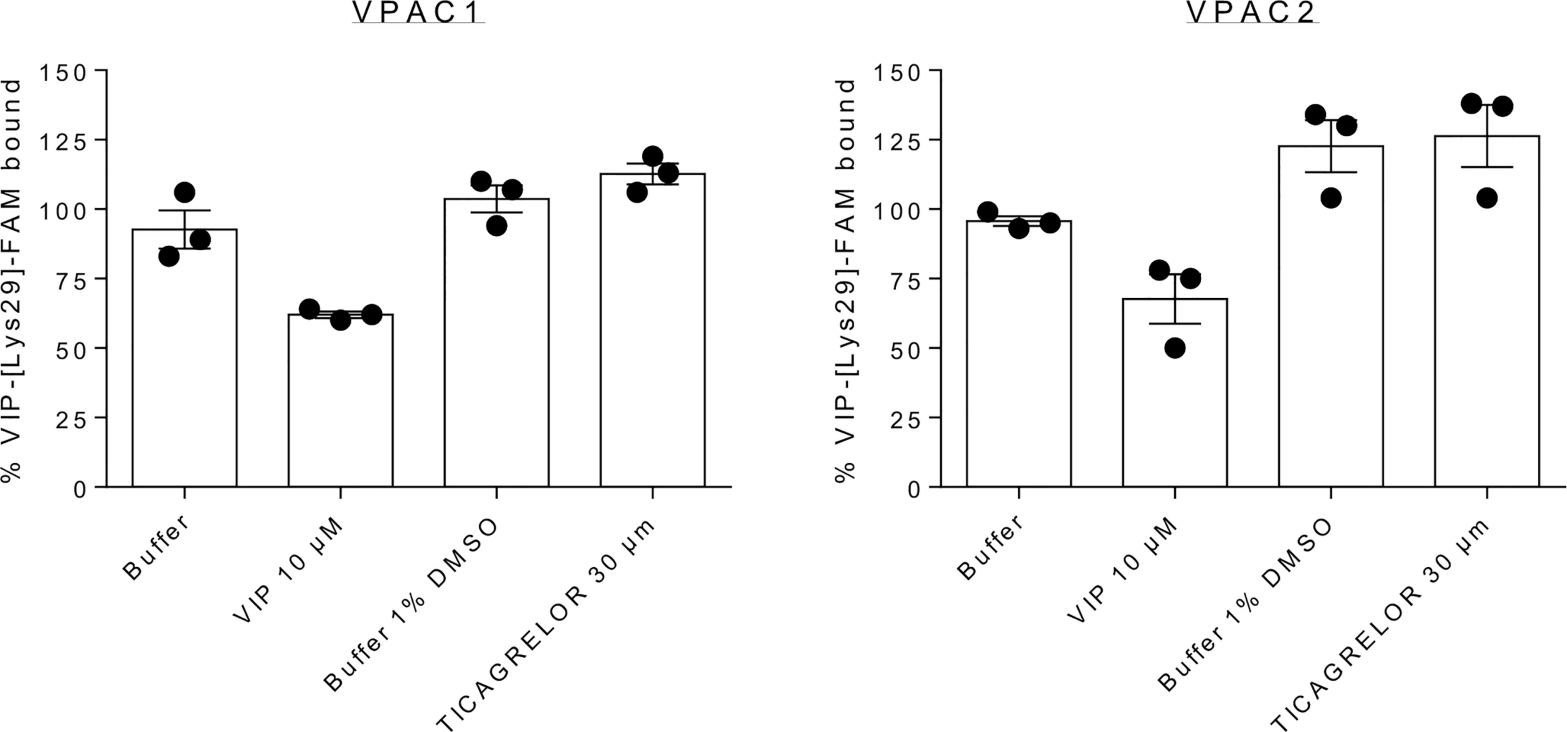
Dissociation of VIP-[Lys^29^]-FAM from complex formed after 30 min. incubation at 20°C with CHO cells expressing VPAC1 or VPAC2. Measured at 60 min., VIP-[Lys^29^]-FAM remained bound after addition of 30 µM ticagrelor or corresponding buffers but dissociated after addition of 10 µM VIP – an endogenous orthosteric agonist. The presented results are the means ± SEM of 3 independent experiments performed in duplicate.

In addition to the above discussion on the state-of-art of GPCR structure determination, we observed that even if ticagrelor behaves as weak modulator of VPAC receptors, it is rather more efficient in reducing VPAC receptors activity than to modify VIP binding. Indeed, to investigate the binding mode of ticagrelor further experimentally, we performed kinetics experiments of the tracer dissociation. As shown in **Figure 4**, addition of 10 µM VIP at steady state induced a rapid dissociation as compared to buffer alone. In the presence of 30 μM ticagrelor, the dissociation of the tracer was very slow and comparable to the kinetics observed in the presence of buffer only. These results suggest that the binding site of ticagrelor is distinct from the orthosteric binding site of VPAC receptors as it did not compete with the tracer like VIP did. Although more detail experiments could be performed here, weak stability of ticagrelor complexes in concentrations greater than 30 μM prevented us from a thorough optimization of conditions. Nevertheless, the orthosteric binding mode of ticagrelor, similar to the VIP binding mode, was rejected based on these preliminary results.

In **Supplementary Figures S6–** **S8** we presented discarded, alternative binding modes of ticagrelor to orthosteric TMD and ECD binding sites. Yet, as we mentioned above, these alternative binding modes of ticagrelor seem much less probable as none among class B GPCR structures with a small molecule orthosteric antagonist has been released in PDB so far (50).

**Figure 5:**
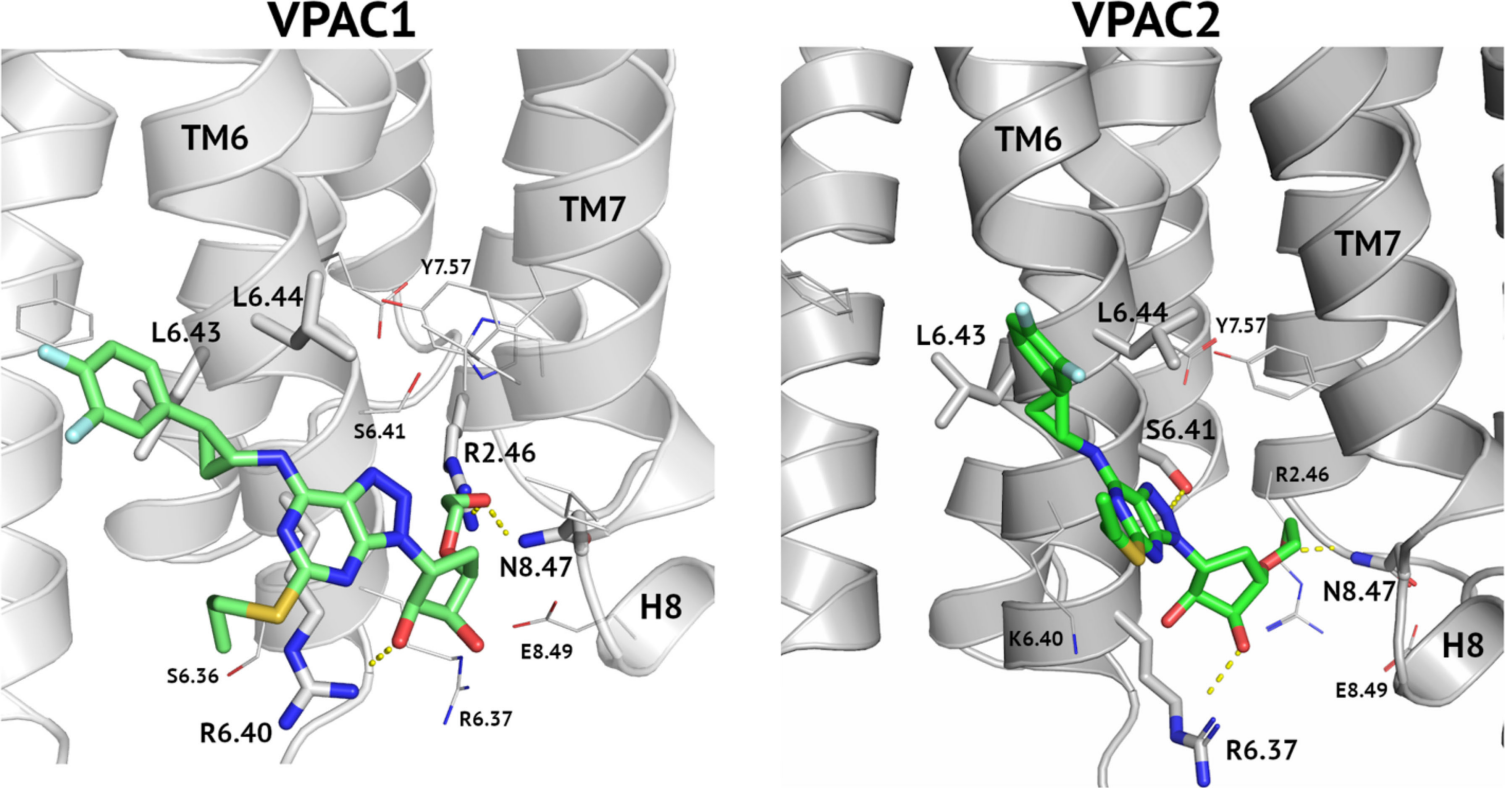
An allosteric binding mode of ticagrelor to VPAC1 and VPAC2. A hydroxyethyl group interacts with Arg2.46 and Asn8.47 and thus blocks the receptor activation. A hydrogen bond between a hydroxyl group of a cyclopentyl group and Arg6.40 (VPAC1, left) or Arg6.37 (VPAC2, right) additionally stabilize the ligand position. A difluorophenyl group of ticagrelor is located between two adjacent leucine residues of TM6.

Cangrelor, an ATP analogue with the antiplatelet effect like ticagrelor, did not modulate the VIP receptors response in pharmacological assays despite encouraging results of VS (see **Table 2**, **Figure 2** and **Supplementary Figure S1**). In VS against TMD sites (both, orthosteric and allosteric) fitness of both, cangrelor and ticagrelor were comparable. In VS against ECD cangrelor demonstrated better fitness to the receptor cavity than ticagrelor. The latter contradicts with experimental results which showed that ticagrelor is a more potent inhibitor of VPAC receptors than cangrelor. Based on this, the ECD binding site had to be excluded because it would favour cangrelor. Nevertheless, limitations of VS prevented us to discard any of the TMD binding sites based on it. A most probable reason for high scores of cangrelor in VS is the negatively charged, deprotonated phosphoryl chain of cangrelor. Such functional group obviously contributes more significantly to the total energy of interactions than all uncharged functional groups of ticagrelor. Namely, this negatively charged phosphoryl chain of cangrelor interacts with positively charged Arg6.37 and Arg6.40 (the allosteric binding mode). Such electrostatic interactions significantly contribute to the interactions energy term included in XP-Gscore (see **Supplementary Figure S5**). Yet, if we inspected allosteric binding modes of these two compounds in detail, we can observe crucial differences between them. Cangrelor remains mostly outside of the small cavity between TM6 and TM7 (and H8) (see **Supplementary Figure S5**). In the opposite, ticagrelor is located inside this cavity and interacts *via* hydrogen bonding with two residues (Arg2.46 and Asn8.47) in it (see **Figure 5**). Only the ticagrelor binding mode resembles known binding modes of NAMs that have been characterized so far either by X-ray crystallography or cryo-EM (49, 60). Such binding modes of NAMs of class B GPCRs ensure interactions with TMD residues that prevent the TM6 bending and moving outwards during the receptor activation.

A similar discussion on VS results can be carried out in the case of the orthosteric binding site (see **Supplementary Figure S6**). If ticagrelor were bound to the orthosteric site of VPAC1 three residues: Asn3.43, Gln7.49 and Lys5.40 would be involved. The latter residue (Lys5.40) is substituted with Arg5.40 in VPAC2 but not in PAC1 (see **Supplementary Figure S7**). Arg5.40 could contribute to better interactions energy in the VPAC2-ticagrelor complex than Lys5.40 in the VPAC1-ticagrelor complex and this is also in agreement with bioassays results (see below: *VPAC1/VPAC2/PAC1 receptor subtype selectivity of ticagrelor*). But we cannot discard the cangrelor binding mode in this case and thus explain the bioassays results because cangrelor could also interacts with Arg/Lys5.40 in such binding mode (see **Supplementary Figure S6**, right). As the described above VS results only partially could confirm the allosteric binding site or discard the orthosteric one we decided to perform additional MD simulations of VPAC2 complexes (see below).

### VPAC1/VPAC2/PAC1 Receptor Subtype Selectivity of Ticagrelor

The described above allosteric binding mode of ticagrelor was confirmed in molecular dynamics simulations of inactive VPAC1, VPAC2 and PAC1 receptor systems. We used 100 ns MD simulations to elucidate molecular basis of the VPAC1/VPAC2/PAC1 receptor sub-type selectivity for ticagrelor observed in pharmacological tests. Although ticagrelor demonstrated inhibitory effect on both VPAC receptors, the highest response in VIP binding assays was observed for VPAC2. A similar conclusion was drawn from MD results (see below).

Conformational fluctuations of TMD stabilized after about 20 ns of production runs with the heavy atom backbone RMSD equal to about 3.5 Å (see **Supplementary Figure S4**). The overall ligand position was kept in all cases – VPAC1, VPAC2, and PAC1 simulation systems with maximal RMSD values of 5 Å, 4 Å, 6 Å for VPAC1, VPAC2, PAC1, respectively (see **Figure S9**). At the end of simulations, the ligand heavy atom RMSD was in the range of 2-3 Å ± 0.5 (see **Supplementary Figure S9**). The least fluctuations of the ligand position were observed in the case of VPAC2 suggesting the highest affinity of ticagrelor to this receptor sub-type.

To further confirm the allosteric binding mode of ticagrelor we performed longer, 200 ns MD simulations for one ticagrelor complex of the highest affinity (VPAC2). Again, the least fluctuations of the ligand position were observed for the allosteric binding mode (see **Supplementary Figure S10** and **Figure 5**, right). The orthosteric binding mode proposed by VS changed rapidly during the first 20 ns of the simulation (RMSD values exceeded 10 Å), in contrast to the stable position of ticagrelor in the allosteric site (RMSD values fluctuated around 3 Å – see **Supplementary Figure S10**). The proposed interaction between ticagrelor and the orthosteric site residue Arg5.40, which was observed in VS, was not preserved in this MD-refined binding mode of ticagrelor. Thus, there could be no explanation for VPAC1/VPAC2 subtype selectivity of ticagrelor observed in the bioassays results based on such orthosteric binding mode of it. In contrast, the allosteric binding mode (see **Figure 5**, right) could easily explain the VPAC1/VPAC2 subtype selectivity of ticagrelor (see below).

**Figure 6:**
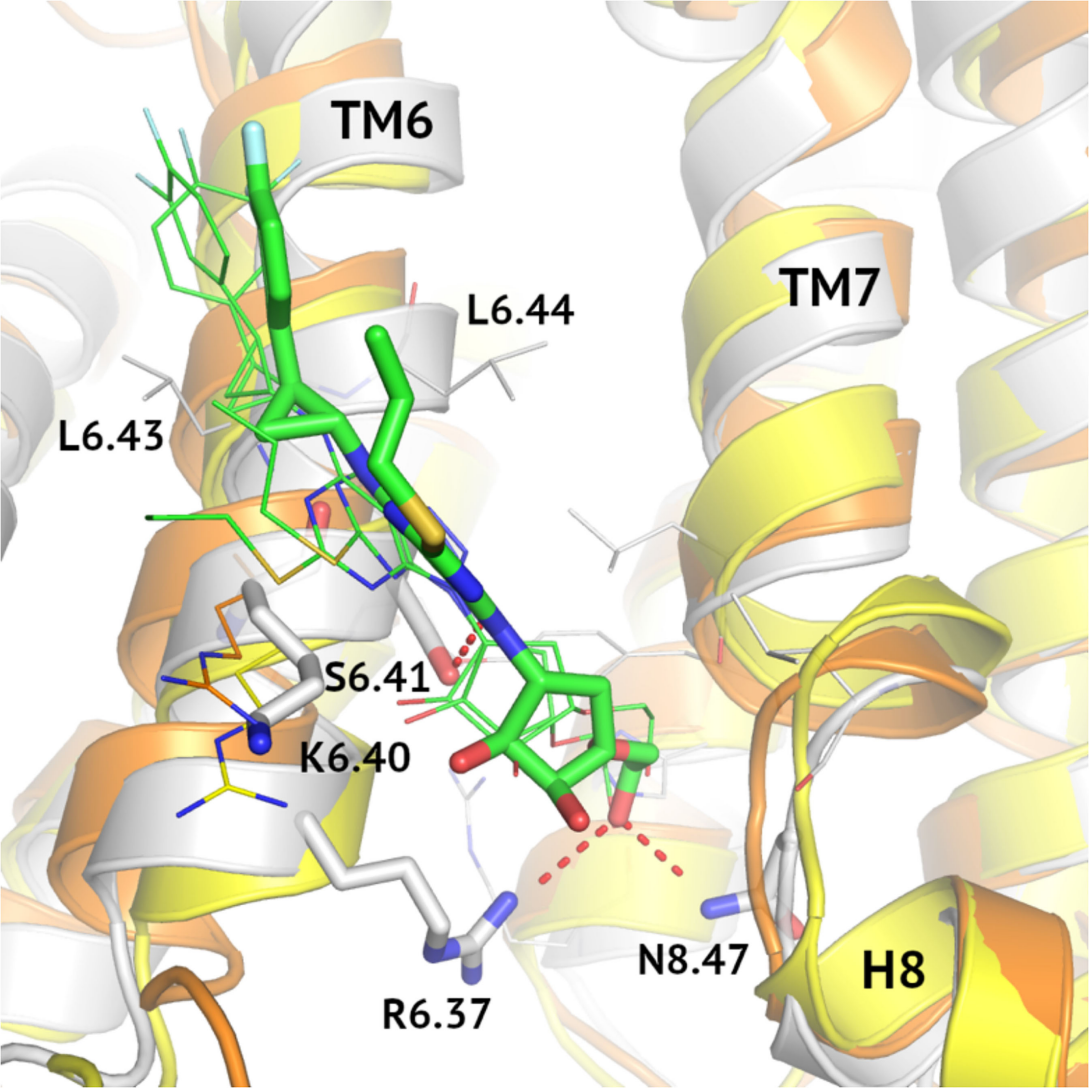
MD-refined allosteric binding modes of ticagrelor to VPAC1/VPAC2/PAC1. MD simulations of VPAC1/VPAC2/PAC1 complexes demonstrated the impact of the R6.40/K6.40 mutation in the NAM-binding region on receptor interactions with ticagrelor (green). The VPAC2-ticagrelor complex was shown in grey, VPAC1-ticagrelor – in orange, and PAC1-ticagrelor – in yellow. Hydrogen bonds in the VPAC2-ticagrelor complex were marked in red and ticagrelor was depicted with sticks. Here, only R6.40/K6.40 were shown in detail in all three receptors because other binding site residues were the same.

MD results for the allosteric site differentiating VPAC2 from VPAC1 and PAC1 can be explained by sequence differences of these receptors in this region (see **Figure 6**). Although the relative orientation of ticagrelor is the same in all cases, Arg6.40/Lys6.40 mutation causes differences in its detail binding modes for these three receptors. In the case of VPAC1 and PAC1 Arg6.40 interacts with the pentose ring of ticagrelor while substitution Lys6.40 in VPAC2 weaken such electrostatic interaction. As a result, ticagrelor is free to move closer to the cavity between TM6 and TM7/H8 and to form interactions with Ser6.42, Arg6.37 and Asn8.47. Thus, a network of interactions in the highly conserved area of His2.50 (61) in VPAC2 could be stabilized in its inactive state in a slightly better way than in the case of VPAC1 and PAC1. Notably, interactions between ticagrelor and Ser6.41 in VPAC2 were refined during the MD simulation resulting in a hydrogen bond stabilizing the ligand binding. A similar hydrogen bond between Ser6.41 and NAM was also observed in other, known so far, class B structures, e.g., the crystal structure of GLP-1R (PDB ID: 5VEW). What is more, Ser6.41 is a conserved residue that is present in other class B GPCRs (38), except CRFR1, CRFR2, and CRLR.

Another fragment of ticagrelor refined in MD was a difluorophenyl group (compare the VPAC1 complex in **Figure 5**, left – before MD, and 6 – after MD refinement). This group adjusted its position to fit the cavity between two adjacent Leu6.43 and Leu6.44 in TM6. Again, in the case of VPAC2 the mutual adjustment of the difluorophenyl group and valine residues seemed to be slightly better than in the case of VPAC1 and PAC1 (see **Figure 5** - right, and **Figure 6**).

Interestingly, our previous mutagenesis studies pointed out that Arg6.37 and Arg/Lys6.40 contribute differently to VPAC1 and VPAC2 activity. Indeed, mutation into alanine of Arg6.37, Arg6.40 or both in VPAC1 did not alter the VIP binding (62). While mutation of the corresponding amino acids in VPAC2 increased up to 19-fold VIP IC50 values, thus reflecting a decreased affinity of VIP for the mutated receptors (63). Similarly, we found that in VPAC1 only the Arg6.37Ala mutant had a significant reduction in the maximal VIP capacity to increase intracellular calcium, the Arg6.40Ala mutant being undistinguishable from the wild-type receptor. But in VPAC2, both Arg6.37Ala and Lys6.40Ala mutants displayed a significant reduction in the maximal VIP capacity to increase intracellular calcium. No intracellular calcium increase was observed following VIP stimulation of the double Arg6.37Ala and Lys6.40Ala mutant. These results highlight the more important contribution of Arg6.37 in stabilizing the inactive state of VPAC receptors comparing Arg6.40 (Lys6.40) and thus, indirectly also confirm the proposed allosteric binding mode of ticagrelor.

## Conclusions

In the presence of difficulties in drug discovery for VIP and PACAP signalling pathways we presented an alternative approach based on the drug repositioning concept. We demonstrated that activity of VPAC1/VPAC2/PAC1 receptors can be modulated by already registered drugs which significantly accelerates the drug discovery process. We discovered a novel drug-target interaction involving ticagrelor, a P2Y12 receptor antagonist, and VIP/PACAP receptors. Intriguingly, cangrelor, a P2Y12 receptor antagonist closely related to ticagrelor, did not evoke a similar response on VPAC receptors, possibly due to a negatively charged phosphoryl group. To explain reasons for lack of cangrelor activity additional experiments with other, similar compounds could be performed, e.g., compounds with a cangrelor phosphoryl group substituted by an uncharged hydroxymethyl group (CID: 21847525) or an ethyl group (CID: 148526362). However so far, this negative feedback about cangrelor has already enabled us to elucidate molecular basis of ticagrelor interactions with VPAC1/VPAC2/PAC1 receptors and to propose its mechanism of action as a negative allosteric modulator of these receptors. MD simulations of ticagrelor complexes in the explicit POPC/cholesterol membrane environment provided explanation of its different rates of cellular response comparing VPAC1 and VPAC2 receptors. Namely, differences in the VPAC2 response to ticagrelor comparing VPAC1 and PAC1 are mostly due to the Lys6.40/Arg6.40 sequence variance in the NAM-binding region of VIP and PACAP receptors. Although the allosteric binding mode of ticagrelor has not been fully confirmed experimentally due to its low potency and solubility, our results are consistent with our hypothesis. Nevertheless, these results provide basis for future drug design of any sub-type selective VPAC1/VPAC2/PAC1 active compounds. To our knowledge, this is the first time that a potential negative allosteric modulator of the VIP and PACAP receptors has been identified.

## Methods

### Structure-Based Virtual Screening

At the time of the beginning of this study, cryo-EM structures of class B GPCRs with positive allosteric modulators (50, 64) were not known, so we have not included them in virtual screening. Following our recent results described elsewhere (41) TMD and ECD regions forming the orthosteric binding site were treated separately in VS. Namely, we screened ZINC15 compounds with the VIP-binding orthosteric site of VPAC1 divided into the TMD part and the ECD part. In both cases, the VPAC1 homology model based on the GLP-1R structure (PDB id: 5VEW) was used. This VPAC1 model, generated with GPCRM (35–37), was validated in the enrichment study (38, 39) and described in detail (TMD and ECD) in our previous work (41).

Binding sites were selected based on the center of mass of ligands in known PDB structures of class B GPCRs. Namely, the allosteric site was defined based on the location of the PF-06372222 modulator in the GLP-1R structure [5VEW (49)]. The location of the orthosteric site in both, treated separately in VS parts (TMD and ECD), was defined based on the location of glucagon-like peptide 1 agonist in the cryo-EM structure of GLP-1R [5VAI (65)]. For definition of the orthosteric site in the ECD part of the receptor we additionally used SiteMap, which was described in (41). The docking box size was set to 30 Å. VS was performed with the Glide extra precision mode (XP) with standard settings described in (38, 39). Out of 5508 compounds retrieved from ZINC15 (66) (world-approved drugs, accession date: Sept 2017, including ambiguous entries, e.g., different accession numbers but the same compound) 380 compounds failed automatic conversion by LigPrep (hydrogen atoms adding, partial charges assignment) and remaining 5128 compounds were converted by LigPrep to 6977 ligands used for VS. Additional 1849 ligands represented additional low-energy conformations generated by LigPrep for ZINC15 compounds. XP-VS provided 6884 (TMD-allosteric), 6965 (TMD-orthosteric), and 6932 (ECD-orthosteric) ligand poses. Many compounds were twice, or three times included in these VS data sets, either because of a larger number of possible low-energy conformers generated by LigPrep, or because ZINC15 included ambiguous entries for some compounds. For example, the ZINC15 data set included two entries for rebapimide: ZINC000009330880 and ZINC000009330879.

Results were filtered against PAINS (66) and REOS (67) data sets using Maestro. Maximum 150 top compounds sorted by XP-Gscore values were selected for further analysis. Compounds with high molecular masses (>500g/mol) were discarded. Also, compounds such as: sucrose, acarbose, lactulose, maltotetraose, glucuronide, L-tryptophan etc. were discarded as false positives. Finally, we selected top 150, 123, 141 compounds from VS against ECD (ortho), TMD (ortho) and TMD (allo), respectively. The above data sets were subjected to manual analysis to categorize them in drug classes (see **Supplementary Tables S1**, **S2**) and to select several compounds for pharmacological testing. After careful analysis, we decided to test six representative compounds in bioassays (see **Table 1**).

### Cell Lines

CHO-WTA11 cells, co-expressing apoaequorin and Gα16, were cultured in Ham’s F12 medium (Thermo Fisher Scientific) supplemented with 10% fetal bovine serum (FBS, Thermo Fisher Scientific), 100 U/ml penicillin, 100 µg/ml streptomycin (Thermo Fisher Scientific) and 250 µg/ml zeocin (InvivoGen) at 37°C with a constant supply of 5% CO_2_. Cell lines expressing human VPAC1 and human VPAC2 receptors were described previously (68).

### VIP Binding Assays

We used the FACS analysis with VIP-[Lys^29^]-FAM (VIP-[Lys^29^][5(6)-Carboxyfluorescein, JPT Peptide Technologies GmbH] as a tracer. For competition experiments, VPAC1 or VPAC2 expressing CHO cells were incubated for 1 h at 4°C in 100 µl cold FACS buffer (PBS, 0.1% BSA, 0.1% sodium azide) containing 10 nM FAM–labelled VIP and 30 µM of small molecules (cangrelor, flupiritine, midodrine, rebapimide, tenofovir, ticagrelor, zanamivir (Sigma)). For kinetics experiments, the cells were incubated for 30 minutes at 20°C in 100 µl cold FACS buffer containing 10 nM FAM–labelled VIP. At equilibrium, 10 µM VIP, 30 µM ticagrelor or 1% DMSO were added to the samples and incubated for up to one more hour. After addition of 2 ml cold FACS buffer, the cells were harvested by centrifugation (560 g, 4°C, 4 min), resuspended in cold FACS buffer and fluorescence was evaluated by FACS. The fluorescence level was analysed using a FACS Gallios flow cytometer (Becton Dickinson) and the median cell fluorescence (MCF) intensity was determined. Non-specific binding was determined *via* MCF in the presence of 3 µM unlabelled VIP (Bachem).

### Aequorin-Based Calcium Mobilization Assay

Calcium mobilization was measured in CHO cells expressing VPAC1 or VPAC2 by an assay based on the luminescence of mitochondrial aequorin as previously described (68). Briefly, cells were collected from plates with 5 mM EDTA in PBS, pelleted, re-suspended at a density of 5.10^6^ cells/ml in DMEM/Ham’s F12 (Invitrogen) supplemented with 0.1% BSA and incubated with 5 µM coelenterazine H (Promega) for 4 h at room temperature under gentle agitation in the dark. Cells were then diluted to a density of 10^6^ cells/ml and incubated for one more hour. For the agonist assay, 50 µl of cell suspension were added to microplate wells containing the small molecules diluted in a volume of 50 µl DMEM-F12. Calcium increase was evaluated by measuring for 30 seconds the luminescent signal (integration of area under the curve) resulting from the activation of the aequorin-coelenterazine complex using a Centro LB 960 luminometer (Berthold Technologies). The data were normalized for basal (0%, background removal) and maximal luminescence (100%) corresponding to the signal measured following exposure to 20 µM ATP. For the antagonist assay, 25 µl of cell suspension were added to 25 µl of tested compounds and incubated for 30 min at room temperature, then 50 µl of VIP (3 or 10 nM) or PACAP-38 (10 or 30 nM) solution were added, and the luminescent signal (integration of area under the curve) was recorded for 30 sec in a Centro LB 960 luminometer (Berthold Technologies). The data were normalized for basal (0%, background removal) and maximal luminescence (100%) corresponding to the signal measured following exposure to of VIP or of PACAP-38 alone.

### Molecular Dynamics Simulations

Complexes of ticagrelor and VIP/PACAP receptors were prepared with Maestro (69) and CHARMM-GUI (70), like previously in (38, 39, 41). POPC lipids with cholesterol molecules with ratio of 3:1 formed lipid bilayers surrounding receptor complexes. Receptor complexes with membrane embedded were solvated with TIP3P with ionic concentration (Na^+^, Cl^-^) of 0.15 M. A total number of atoms of simulation systems was ca. 97000. Charmm36 was used in each MD simulation. An equilibration stage, following the first 2 ps of conjugate gradient minimization, included six steps, lasting for: 25 ps, 25 ps, 25 ps, 50 ps, 50 ps, 50 ps. During six equilibration steps atomic position restraints were gradually released, e.g., for protein backbone atoms a force constant of a harmonic potential decreased from 10 (1st step), 5.0, 2.5, 1.0, 0.5, to 0.1 kcal·mol^−1^·Å^−2^ (6th step). Only for the first step of equilibration, a 1 fs time integration step was used. The first two steps were performed in NVT (Langevin dynamics), the next four in NPT [1 bar, 303.15 K, Nose-Hoover Langevin piston (71, 72)]. The production run was performed in NPT (1 bar, 303.15 K, Nose-Hoover Langevin piston) and lasted 100 ns in each case. The GPU version of NAMD was used for all simulations (73, 74).

## Data Availability Statement

The original contributions presented in the study are included in the article/**Supplementary Material**. Further inquiries can be directed to the corresponding author.

## Author Contributions

All authors contributed to the article and approved the submitted version. Conceptualization: DL. Methodology: DL, IL. Software: DL. Validation: DL, IL. Formal analysis: DL, IL. Investigation: DL, IL. Data Curation: DL. Virtual screening and molecular dynamics: DL. Assigning of drug classes: IL. Bioassays: IL. Writing—original draft preparation: DL, IL. Writing—review and editing: DL, IL. Visualization: DL, IL. Supervision: DL. Project administration: DL. Funding acquisition: DL, IL.

## Conflict of Interest

The authors declare that the research was conducted in the absence of any commercial or financial relationships that could be construed as a potential conflict of interest.

## Publisher’s Note

All claims expressed in this article are solely those of the authors and do not necessarily represent those of their affiliated organizations, or those of the publisher, the editors and the reviewers. Any product that may be evaluated in this article, or claim that may be made by its manufacturer, is not guaranteed or endorsed by the publisher.

## Abbreviations

NAM, negative allosteric modulator; PAM, positive allosteric modulator; GPCR, G protein-coupled receptor; ECD, extracellular domain; TMD, transmembrane domain; Cryo-EM, cryogenic electron microscopy; VIP, vasoactive intestinal peptide; PACAP, pituitary adenylate cyclase activating polypeptide; PAR-2, protease activated receptor 2; CRLR, calcitonin receptor-like receptor; ADP, adenosine diphosphate; MD, molecular dynamics; FACS, fluorescence-activated cell sorting; CHO cells, Chinese hamster ovary cells; DPP-4, dipeptidyl peptidase 4; COX-2, cyclooxygenase-2; NMDAR, N-methyl-D-aspartate receptor; RT, reverse transcriptase.
