# Supplementary material for: Drug Repositioning For Allosteric Modulation of VIP and PACAP Receptors
Source: Front Endocrinol (Lausanne). 2021 Nov 18;12:711906. doi: 10.3389/fendo.2021.711906 (PMC8637020; doi:10.3389/fendo.2021.711906)
Supplement: Supplementary file 1 [file DataSheet_1.pdf]

Supplementary

Drug repositioning for allosteric modulation of VIP and PACAP receptors.

Ingrid Langer[1], Dorota Latek[2,*]

[1]Institut de Recherche Interdisciplinaire en Biologie Humaine et Moléculaire (IRIBHM), Faculty of Medicine, Université libre de Bruxelles, B-1070 Brussels, Belgium

[2]Faculty of Chemistry, University of Warsaw, 02-093 Warsaw, Poland

Figure S1. Results of functional assays – ticagrelor and cangrelor. Results of calcium mobilization assays for ticagrelor and cangrelor tested in various concentrations up to 30µM in CHO cells expressing VPAC1 (top panels) or VPAC2 (bottom panels).

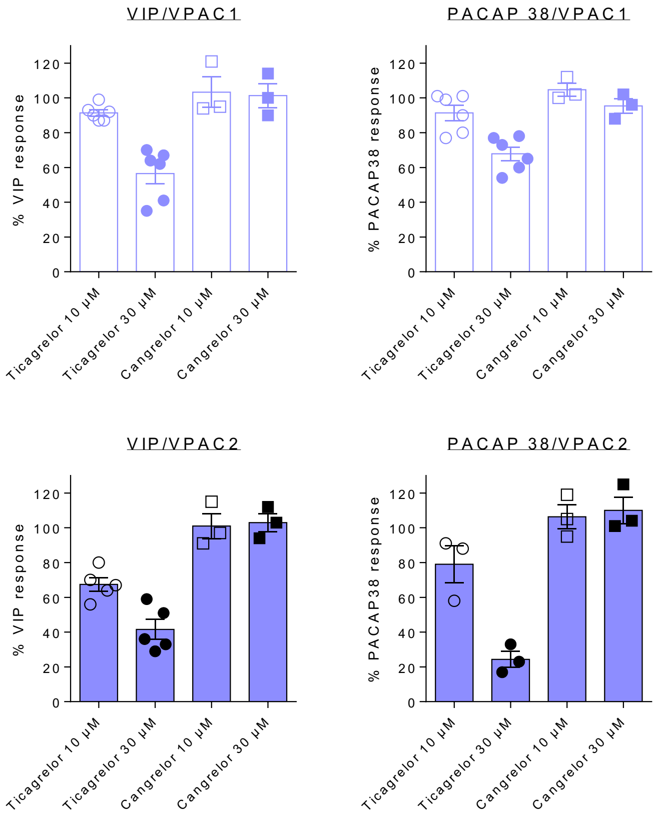

Figure S2. Results of binding assays – selected compounds. Results of VIP competition binding assays in CHO cells expressing VPAC1 or VPAC2 for compounds selected from virtual screening.

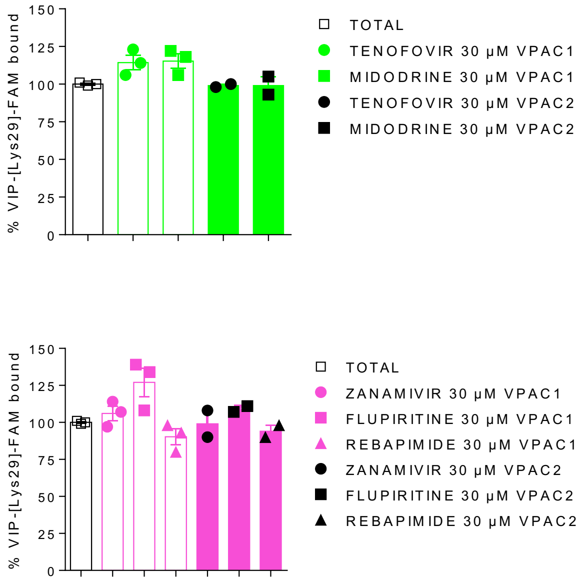

Figure S3. Results of functional assays – selected compounds. Results of calcium mobilization assays for compounds selected in virtual screening in various concentrations up to 30µM in CHO cells expressing VPAC1 or VPAC2.

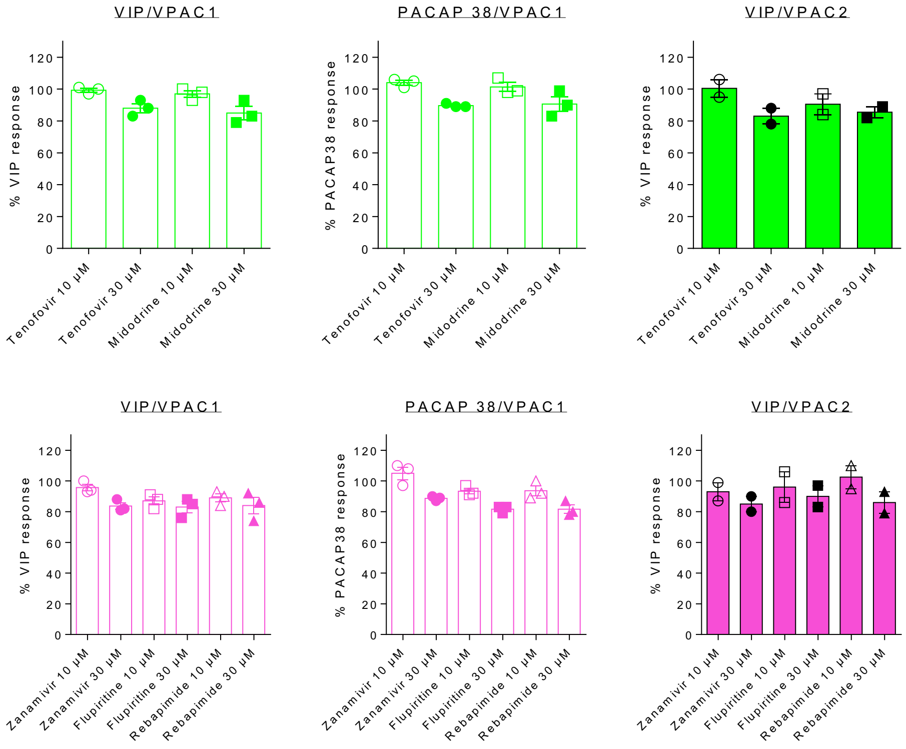

Figure S4. Results of MD simulations. VPAC1 receptor backbone RMSD referring to the VPAC1 homology model.

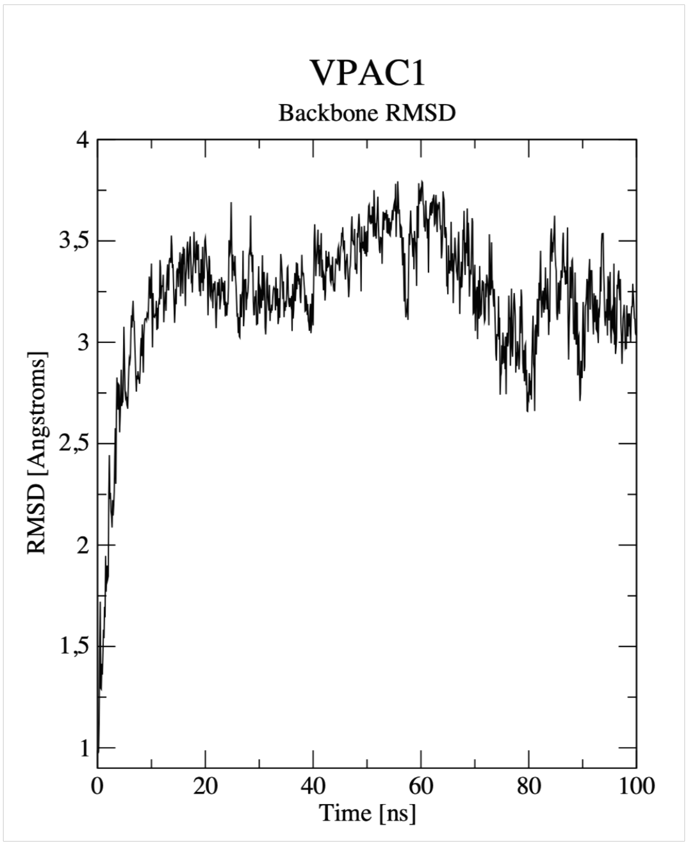

Figure S5. NAMD-binding allosteric site – cangrelor. An allosteric binding mode of cangrelor in the TMD allosteric site of VPAC1 involving R6.40 that was obtained in virtual screening. Noteworthy, cangrelor did not induce noticeable response on VPAC receptors in any tested bioassay.

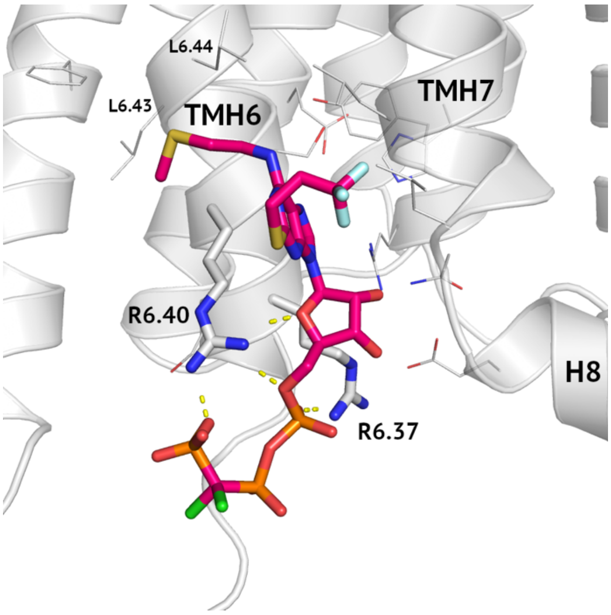

Figure S6. Peptide-binding orthosteric TMD site – ticagrelor and cangrelor. Less probable, alternative binding modes for ticagrelor (left) and cangrelor (right) for the TMD orthosteric binding site of VPAC1 that were obtained in virtual screening. In contrast to ticagrelor, cangrelor did not induce noticeable response on VPAC receptors in any tested bioassay but this can be hardly explained by comparing the orthosteric binding modes of these two compounds as presented below.

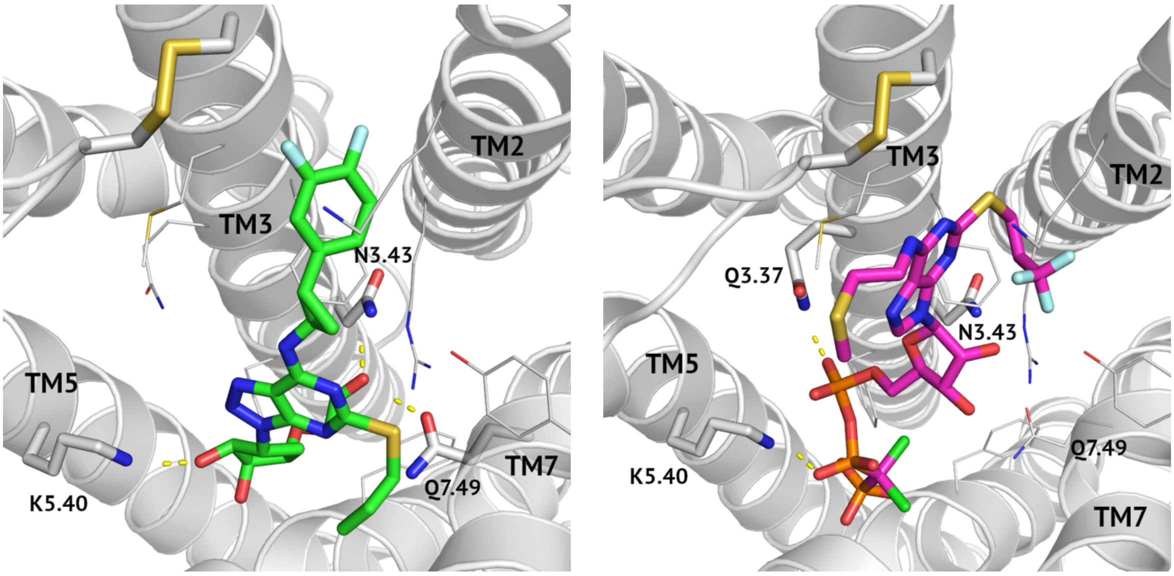

Figure S7. Peptide-binding orthosteric TMD site – sequence diversity of VIP and PACAP receptors. VPAC1 (orange) and PAC1 (yellow) share most of binding site residues, while VPAC2 (grey) includes: S3.32 (in contrast to non-polar A3.32 in VPAC1 and V3.32 in PAC1), L3.33 (in contrast to polar M3.33), L3.36 (in contrast to aromatic F3.36) and R5.40 (in contrast to K5.40 in VPAC1 and PAC1). Only residue H3.37 is different in PAC1 with respect to VPAC receptors (Q3.37). Ticagrelor was shown in green. None of the described above residues formed direct interactions with ticagrelor in VS. VPAC1, VPAC2 and PAC1 share the same motif CWD (not shown) of the extracellular loop 2 (ECL2) in the region close to ticagrelor.

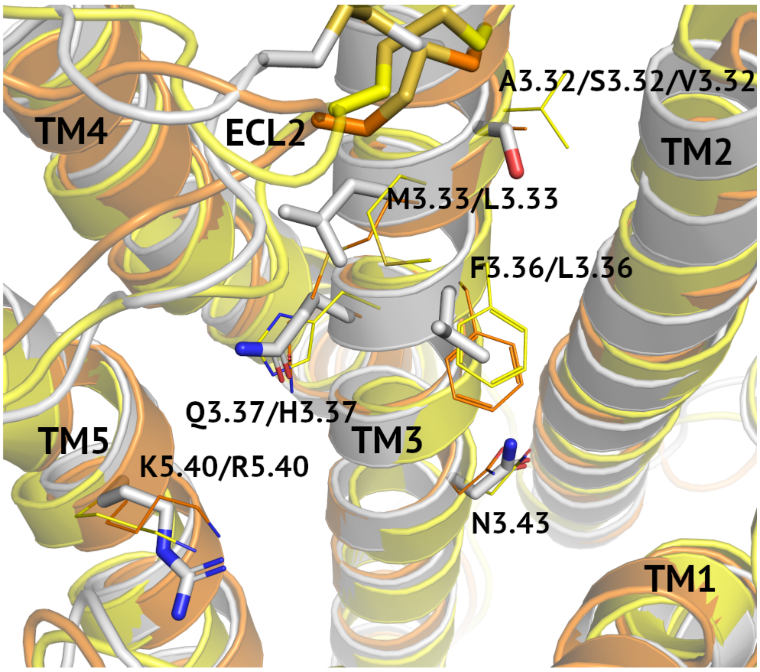

Figure S8. Peptide-binding orthosteric ECD site – ticagrelor and cangrelor. The least probable, alternative binding modes for ticagrelor (left) and cangrelor (right) for the ECD orthosteric binding site obtained in virtual screening. In both cases, two tyrosine residues Tyr39 and Tyr118, forming a two-tyrosine gate in a closed conformation, and Thr71 described elsewhere (Latek et al. IJMS 2019) were depicted.

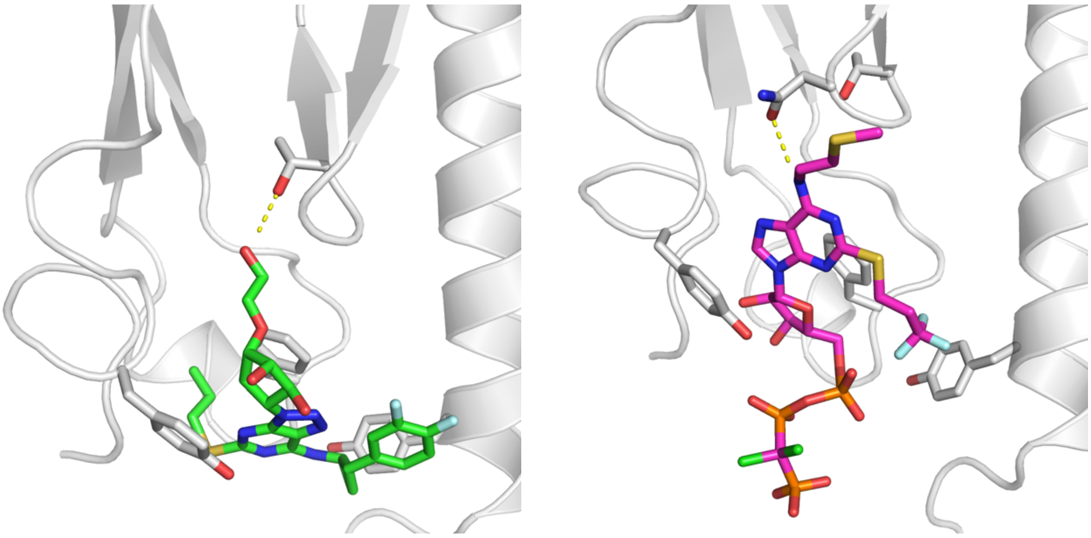

Figure S9. Results of MD simulations. VPAC1/VPAC2/PAC1 complexes with ticagrelor in the TMD allosteric binding site. Ligand (ticagrelor) heavy atom RMSD referring to the screening-derived ligand pose were shown for each receptor complex. Least fluctuations of the ticagrelor position were observed in the case of VPAC2 (middle).

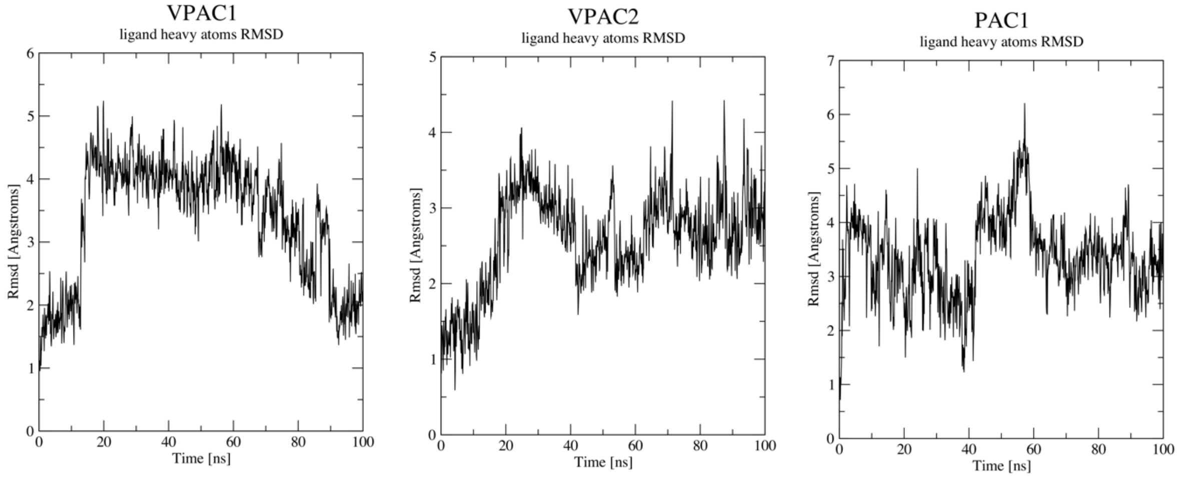

Figure S10. Results of 200 ns MD simulations. Here, we compared two possible binding sites of ticagrelor: allosteric (left) and orthosteric (right). Ticagrelor docked to the orthosteric site of VPAC2 rapidly changes its binding mode in contrast to the stable allosteric binding mode (left). Ligand heavy atom RMSD referring to the screening-derived ligand pose were shown for the allosteric receptor site (left) and the orthosteric receptor site (right).

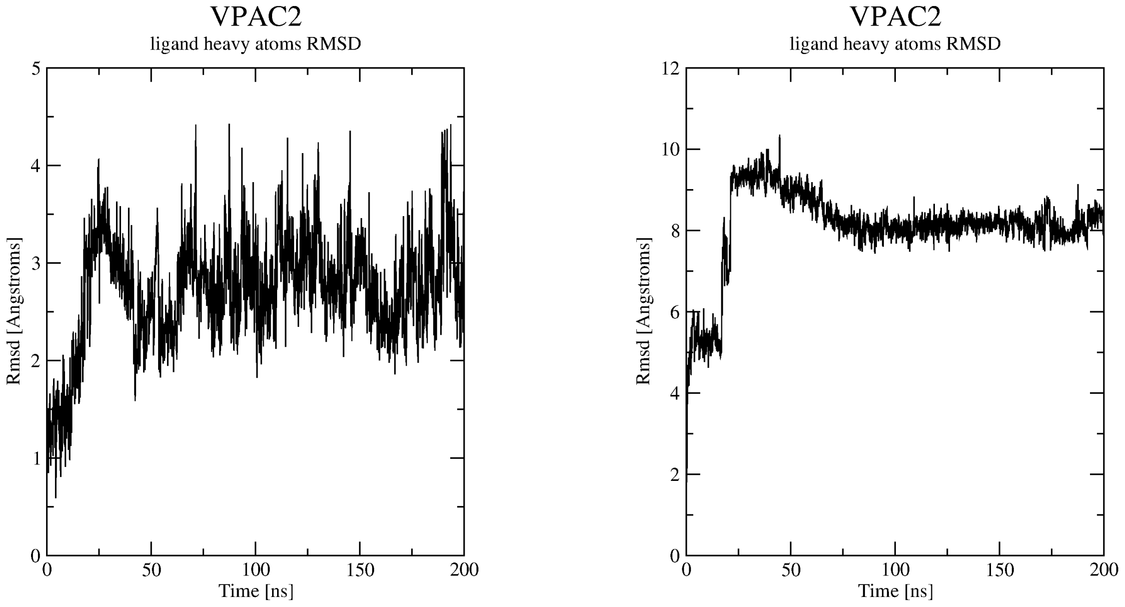

Figure S11. Competition binding curves for VIP (left panel) and ticagrelor (right panel) in CHO cells expressing VPAC1 or VPAC2.

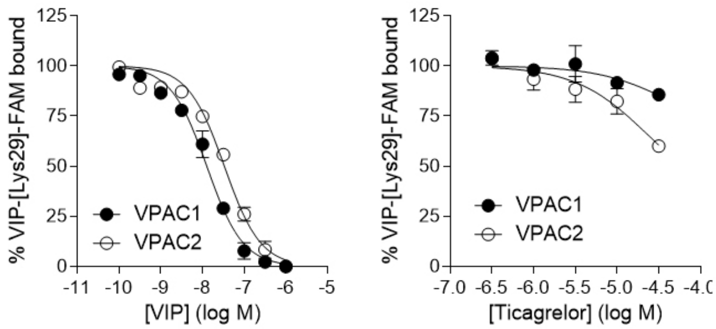

Table S1: Example drug classes found in top 150 compounds from VS (orthosteric site – ECD).

Drug class	*Number of compounds included*
Antiviral	8
antibacterial	5
Treatment of venous diseases	1
Tyrosine kinase inhibitor	3
Serotonin receptor agonist	2
Beta-blockers	2
COX activator	1
COX inhibitor (NSAID)	3
HMGCoA inhibitor	3
Imaging product	2
NMDAR antagonist	1
Cholesterol transporter inhibitor	1
GAT1 inhibitor	1
Rhythm regulator	1
fungistatic	1
Anti-histaminic (H1R)	2
Against cough	1
Thrombin inhibitor	1
PPI	2
DNA synthesis inhibitor	1
Local anesthetic	1
SGLT2 inhibitor	1
VMAT2 inhibitor	1
Anticancer drug (antimetabolite)	3
Anti-parasite	1
diuretic	1
Cannabinoid receptor agonist	1
AT1 receptor antagonist	1
barbiturate	1
Endogenous substances	40
Other	57

Table S2: Compounds manually selected from the VS data sets.

ZINC ID	*Name*	*Drug class/ disease associated*	*Binding site used in virtual screening*
ZINC000003977786	Aloin	gastrointestinal	TMD - NAM
ZINC000003977787	Aloin	gastrointestinal	
ZINC000146708406	Cetraxate	gastrointestinal	
ZINC000001543475	Tenofovir	RT inhibitor	
ZINC000000000637	Pirbuterol	Beta-2 AR agonist	
ZINC000000897261	Pirbuterol	Beta-2 AR agonist	
ZINC000000000507	Midrodine	Alpha AR agonist	
ZINC000000057341	Methocarbamol	Muscle relaxant	
ZINC000000899004	Deoxynojirimycin	Alpha-glucosidase inhibitor	
ZINC000003792417	Sacubitril	Neprilysin inhibitor	
ZINC000004340269	Amiloride	Diuretic (ENAC inhibitor)	
ZINC000000001982	Probenecid	Hypouricemic	
ZINC000013818943	Regadenoson	Adenosine A2AR agonist	
ZINC000029416466	Saquinavir	Protease inhibitor	TMD – orthosteric
ZINC000004097309	Fosinopril	ACE inhibitor	
ZINC000014210876	Eluxadoline	Opioid receptor agonist	
ZINC000014879963	Remikiren	Renin inhibitor	
ZINC000003774999	Candoxatril	Neprilysin+ACE inhibitor	
ZINC000000896717	Zafirlukast	Cys-LT receptor antagonist	
ZINC000043100709	Trametinib	MEK inhibitor	
ZINC000028957444	Ticagrelor	$P2Y_{12}$ receptor antagonist	
ZINC000003978654	Bergenin	anti-inflammation	ECD - orthosteric
ZINC000003918138	Zanamivir	Neuraminidase inhibitor	
ZINC000001530579	Carvedilol	Beta-blocker	
ZINC000009330880	Rebamipide	COX activator	
ZINC000000015515	Zolmitriptan	5HT1R agonist	
ZINC000000607872	Floctafenine	NSAID	
ZINC000000001473	Flupirtine	NMDAR antagonist	
ZINC000001851599	Ridogrel	Anti-thrombotic	
ZINC000003810860	Ezetimibe	NPC1L1 inhibitor	
ZINC000003830276	Benzonatate	Cough suppressant	
ZINC000000001322	Anthralin	DNA synthesis inhibitor	
ZINC000001530811	Tetracaine	Local anesthetic	

ZINC000003819138	Dapagliflozin	SGLT2 inhibitor
ZINC000000001624	Lobeline	VMAT2 inhibitor
ZINC000000897225	Hydroflumethiazide	Diuretic (thiazide)
ZINC000030731219	Hydroxyiminostilbene	Barbiturate
ZINC000000002688	Dolasetron	5-HT3R antagonist
ZINC000052509366	Vemurafenib	BRAF inhibitor
ZINC000013536586	Topiroxostat	Xanthine oxidase inhibitor
ZINC000000538483	Trazodone	SERT inhibitor
ZINC000000001342	Efloxate	Vasodilatator

Table S3. Compounds similar to ticagrelor found in VS data sets (ZINC15 world-approved drugs).

Name	*Drug class / disease associated*
Cangrelor	$P2Y_{12}$ receptor antagonist
Coenzyme A	endogenous
FAD	endogenous
Thioguanosine 5'Diphosphate	immunosuppressive
Fludarabine	anti-cancer
6-Thioguanylic Acid	immunosuppressive
6-Methylthioguanosine Monophosphate	anti-cancer
Clofarabind-5'-Monophosphate	anti-cancer
6-Mercaptopurine Ribonucleoside 5'-Diphosphate	immunosuppressive
Clolar (clofarabine)	anti-cancer
Dpnh (NADH)	endogenous
Sah (S-Adenosyl-L-homocysteine)	endogenous
Olo (Olomoucine)	anti-cancer
